# Dr. Battey’s Case, Etc.

**Published:** 1872-10

**Authors:** 


### DR. BATTEY'S CASE OF CHRONIC CYSTITIS.

In this number will be found a letter of Dr. Battey, in reference to his case of "Cystotomy" published in the June number of the COMPANION. Dr. Battey is a Surgeon of no ordinary ability—as his successful operations in Cystotomy, Ovariotomy, etc., attest.

